# Health inequality of rural-to-urban migrant workers in eastern China and its decomposition: a comparative cross-sectional study

**DOI:** 10.3389/fpubh.2024.1365241

**Published:** 2024-05-13

**Authors:** Sisi Du, Yufan Yang, Miaomiao Zheng, Haiyan Zhang, Tingting Li, Fuman Cai

**Affiliations:** ^1^College of Nursing, Wenzhou Medical University, Wenzhou, China; ^2^The Second School of Medicine, Wenzhou Medical University, Wenzhou, China; ^3^Cixi Biomedical Research Institute, Wenzhou Medical University, Wenzhou, China; ^4^The First Affiliated Hospital of Wenzhou Medical University, Wenzhou, China

**Keywords:** health inequality, concentration index, decomposition, rural-to-urban migrant worker, urban worker

## Abstract

**Objectives:**

As a specific group with high health inequality, it is crucial to improve the health status and health inequalities of rural-to-urban migrant workers. This study aimed to evaluate the health inequality of migrant and urban workers in China and decompose it.

**Methods:**

A cross-sectional study was carried out, using a standardized questionnaire to obtain basic information, self-rated health to evaluate health status, concentration index to measure health inequalities, and WDW decomposition to analyze the causes of health inequalities.

**Results:**

The concentration index of health for migrants was 0.021 and 0.009 for urban workers. The main factors contributing to health inequality among rural-to-urban migrant workers included income, exercise, and age. In contrast, the main factors of health inequality among urban workers included income, the number of chronic diseases, social support, and education.

**Conclusion:**

There were health inequalities in both rural-to-urban migrant and urban workers. The government and relevant authorities should formulate timely policies and take targeted measures to reduce income disparities among workers, thereby improving health inequality.

## Introduction

According to the National Bureau of Statistics (1), China's population mortality rate has decreased from 7.13‰ in 2013 to 7.07‰ in 2020. The average population life expectancy has extended from 67.9 years in 1982 to 76.7 years in 2018 and is expected to reach 79.0 years in 2030 (2). In recent years, with the rapid socioeconomic development and the popularization and promotion of health policies, the mortality rate of the Chinese population has been decreasing, the average life expectancy has been gradually extended, and the health status has been gradually improved (3). However, at the same time, health inequalities between urban and rural residents have become increasingly prominent, and the improvement of the average health level cannot conceal the fact that health inequalities exist. As a social problem that needs to be solved and improved urgently (3–5), health inequalities have attracted the widespread attention of more and more scholars around the world. According to the goals related to the 2030 Agenda for Sustainable Development, reducing social inequalities and health inequalities and ensuring that everyone can have a healthy life is one of the current tasks (6). Confronting health inequalities and reducing them through timely and effective measures is a result of social development, which will have a positive impact on society (7). Based on those pioneering work, most scholars mainly focus on two aspects of health inequality: One is to use the Gini coefficient and the concentration index (CI) to measure and compare (8). Health inequalities are not simply differences in the distribution of health among populations, but differences in the health of populations with additional socioeconomic factors, such as income and education (9). The second is tantamount to investigate the impact of socioeconomic factors such as income, education, and occupation on health outcomes based on the regression model (10). Wagstaff et al. (11) believed that the measurement method of health inequality must be in a position to reflect the economic characteristics of health inequality and be sensitive to socio-economic changes. The concentration index method can measure the health differences caused by different levels of social and economic development, so the concentration index method is favored by most scholars.

Studies have shown that the most intuitive manifestation of the health inequality problem lies in the disparity of health outputs among different populations (12). Many scholars studied health inequalities across regions and populations (12–14). Fan et al. (7) found that 2.4% of health disparities among the older adult population are caused by the uneven development of health levels across provinces, and the main reason for health inequalities across provinces was the uneven access to economic, medical, and educational resources, while at the same time, annual income inequality exacerbated health disparities among the older adult, and those living in less developed regions were more vulnerable to urban vs. rural related health inequalities. Silva-Peñaherrera et al. and Pascual et al. (15, 16) both concluded that there were broad health inequalities among populations in different regions.

Studies have shown that rural-to-urban migrant workers were a specific group with significant health inequalities (17). Compared with other populations, the low standard of living, unstable and hard work, isolation and discrimination due to cultural differences, and weak social support can lead to a series of mental health problems such as depression and suicide among rural-to-urban migrant workers, which seriously endangered their health (9, 18–20). Current domestic and international research on rural-to-urban migrant workers focused on the older migrant worker population. Ma et al. (21) studied health inequalities among urban-urban and rural-urban mobile older adults in China and found that social capital and social integration played an important role in the health of older migrants. Li et al. (3), compared the health disparities between urban and rural older migrant workers and used Fairlie's decomposition analysis to identify the factors influencing health inequalities. However, the aforementioned studies only involved health inequalities of older rural-to-urban migrant workers and urban migrant workers, not all age groups of migrant workers, and were not generalizable. Therefore, this study focused on a group of Chinese rural-to-urban migrant workers and compared them with urban workers to compare the extent of health inequality and to decompose the health inequality. This study may expand the literature on studies related to Chinese rural-to-urban migrant workers. More importantly, it will also provide a reference for the government to develop relevant health policies that can significantly improve the health of rural-to-urban migrant workers and ameliorate health inequalities.

## Methods

### Study design and sampling

Based on differences in economic development and geographical location, China is divided into eastern, central, western and northeastern regions (22). Compared to other regions, the eastern region of China is economically developed (23). Rural-to-urban migrant workers from other regions have flocked to cities in the eastern region to work in order to improve their quality of life and provide better educational environments for their children. Due to China's registered residence policy, the benefits of homestead brought by rural household registration make rural-to-urban migrant workers choose to keep rural household registration to work in cities (24). In addition, industrial enterprises require more workers with medium to low skills, and rural-to-urban migrant workers precisely meet their needs, which further accelerates the career upgrading of urban workers with medium to low skills and reduces the number of urban workers (25). Considering the accessibility of the study participants, cities in the eastern region of China were finally selected as survey sites to obtain specific information on the study population. A cross-sectional survey was conducted in the eastern region of China from August 2019 to January 2020 using a multi-stage stratified sampling method. First, Jiangsu, Zhejiang, Fujian, Guangdong, and Shanghai were randomly selected from 10 eastern provinces and cities. In addition to Shanghai, we also randomly selected Suzhou, Wenzhou, Xiamen and Shenzhen from four other provinces and cities. Next, two districts and 10 communities and corresponding neighborhood committees were selected based on the population size of each of the five cities. Uniformly trained surveyors explained the purpose, method, meaning and precautions for completing the survey to the study participants using a standardized instructional language. After obtaining their consent, the surveyor distributed the questionnaire to them on site and they filled it out by themselves; for those who cannot fill out the questionnaire by themselves, the surveyors filled it out on their behalf according to their answers. Rural-to-urban migrant workers were defined as those with rural household registration who worked in non-agricultural industries in cities for 6 months or more (22), while urban workers were defined as a group with urban household registration who worked in cities. Participants who met the following criteria would be invited to participate in this survey and study: (i) age≥18 years old; (ii) ability to read, write, and communicate in Chinese, and no cognitive impairment. Participants with serious medical conditions and those aged >90 years were excluded.

**Figure 1:**
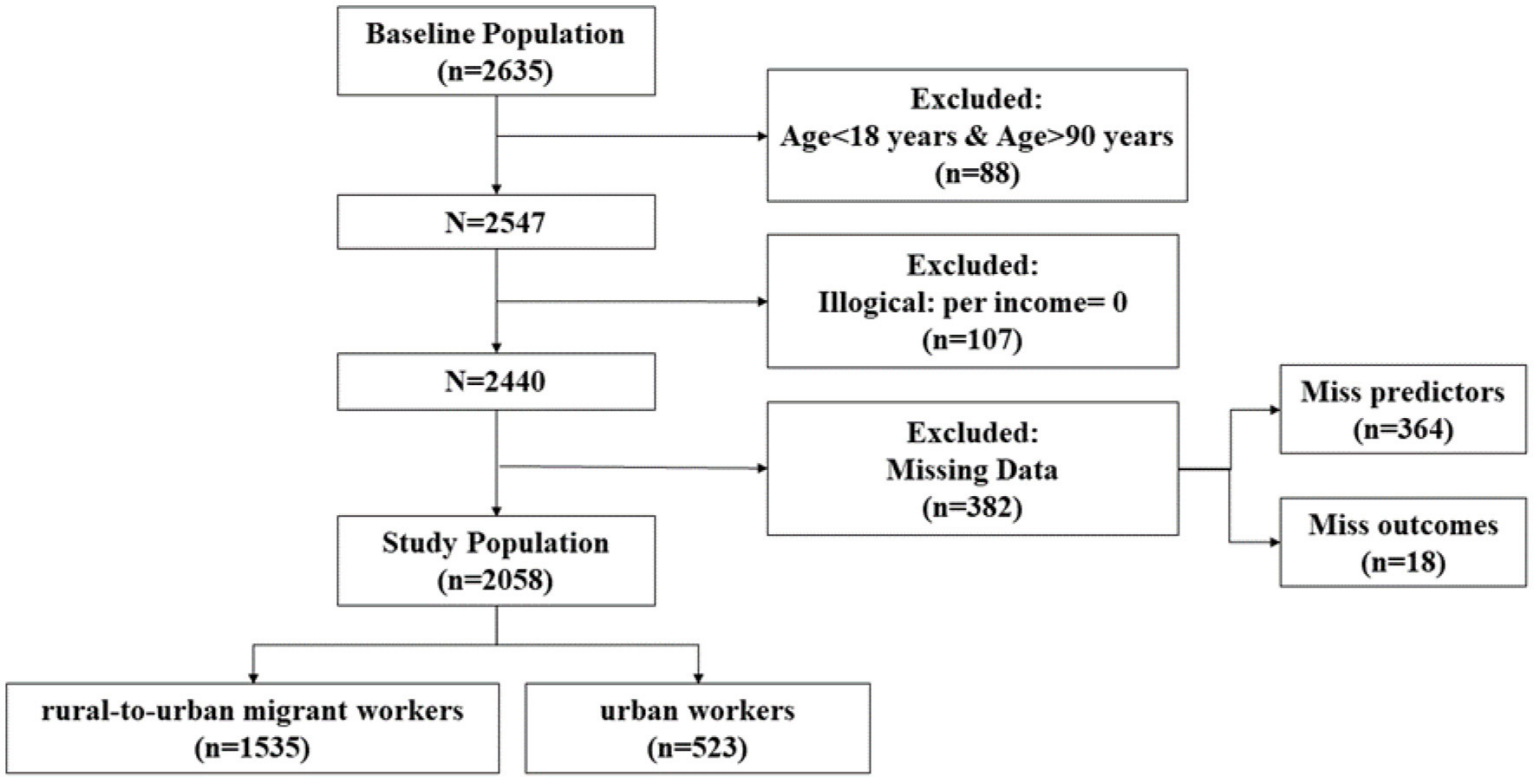
A flow chart for study population selection.

Initially 2,635 people were invited and completed the questionnaire. After eliminating 88 people with invalid age, 107 people with illogicality, and 382 people with missing data, a total of 2,058 people were included, including 1,535 rural-to-urban migrant workers (74.59%) and 523 urban workers (25.41%), as shown in Figure 1.

### Measures

Data were collected from participants using a self-designed standardized questionnaire, including information on six groups of independent variables and health status.

### Independent variables

After reviewing the literature, we found that the results of health inequality should be borne by individuals, but are inseparable from other social subjects, such as families, communities and governments (26–28). Therefore, on the basis of previous studies, from the four aspects of demographic characteristics, social security, family support and health behavior, the independent variables are divided into the following six groups of variables. (1) Socioeconomic factors: As an essential category of health inequality, socioeconomic factors were mainly divided into income [per capita household income (/10,000 CNY)] and education (elementary school, middle school, high school, and high school above). (2) Demographic characteristics: Demographic characteristics included gender (male, female) and age. (3) Living habits: Living habits included weekly exercise (yes, no), smoking duration (never smoking, <1, 1–5, 5–10, and more than 10 years), number of weekly alcohol consumption (never drinking, once, twice, three times, four times, five times, or more) and sleep habits (early to bed and early to wake up, early to bed and late to wake up, late to bed and early to wake up, late to bed and late to wake up). (4) Family factors: Family factors included marital status (never married, married, widowed, divorced, or separated) and pension style (provided by children, savings, government, business pension, and others). (5) Number of chronic diseases: The number of chronic diseases included 0, 1, 2, 3, and 4 or more. (6) Social support: Social support is a score on the Social Support Rating Scale (SSRS). The total score of SSRS ranged from 12 to 66. The higher the score, the better the social support status of the individual, which can be classified as poor social support (score ≤ 22), moderate (score 23–44), and adequate social support (score 45–66) (29, 30). Quantitative variables were directly represented by numerical values. Assign values to categorical variables. In this study, 361 cases were lost variables. The lost samples of pension variables were classified as others and assigned a value of 5. Other variables were largely influenced by individual differences of research objects and were mostly classified variables, so it was unreasonable to fill in missing values through interpolation and fitting methods. Besides, the sample size of this study was large, so direct removal had little impact on research results.

### Health status definition

Since self-rated health is a comprehensive indicator that adequately reflects the health status of an individual and can effectively predict the objective health status of an individual, such as mortality and functional loss (31). Self-rated health was chosen as an indicator to evaluate the level of health in this study. Very good, good, average, poor, and very poor were set as 1, 2, 3, 4, and 5, respectively. Therefore, following the method of Wagstaff et al. (32), this research used the ordered probit model to assign values to self-rated health variables, adjusting them to continuous variables, and converting them to values in the range of [0,1]. The result of the conversion was denoted as SAH.

### Health inequality

The concentration index (CI) and the concentration curve have been widely accepted as a measure of health inequality (33). The CI is closely related to health distribution. When health is evenly distributed among people of different socioeconomic classes, i.e., there is no health inequality, then the CI is 0 and the concentration curve coincides with the diagonal. When the CI is negative, the concentration curve is above the diagonal, it indicates that health is concentrated in the low-income class, i.e., the poor have more health advantages, and there is pro-poor health inequality. When the CI is positive, the concentration curve is below the diagonal, it indicates that the health advantage is concentrated in the high-income class, the health advantage of the rich is more obvious, and there is pro-rich health inequality. The larger the absolute value of the CI, the more serious the health inequality is. The CI is calculated by Equation (1):


(1) $$\begin{array}{l} CI=\frac{2}{H}\mathrm{cov}\left(h_{i},R_{i}\right)=\frac{2}{nH}\overset{n}{\underset{i=1}{\sum }}h_{i}▪R_{i}-1 \end{array}$$

*i* stands for an individual; *h* is for individual health; *H* is the average state of health of the sample; *R* represents the rank of the scores of individuals in the sample, ranked from lowest to highest in income.

Gini coefficient is a commonly used index used internationally to measure the income gap of residents in a country or region. It was first proposed by Corrado Gini, an Italian statistician and sociologist. The maximum Gini coefficient is “1” and the minimum is “0.” The closer the Gini coefficient is to zero, the more equal the distribution of income becomes. The Gini coefficient is too large, indicating that the income gap is still too large, the gap between the rich and the poor is large, and has not yet reached the ideal average level.

Lorenz curve is used to compare and analysis of a country in different age or wealth inequality of different countries at the same time. Using the Lorentz curve, you can visually see the status of income distribution equality or inequality in a country. The degree of curvature of the Lorentz curve is important. Generally speaking, it reflects the degree of inequality in the distribution of income. The greater the curvature, the more unequal the income distribution.

### Decomposition of health inequality

In order to further analyze the causes of health inequality, Wagstaff et al. (11) proposed to divide the CI into components of multiple factors, namely WDW decomposition. The main idea of this decomposition is to separate the factors that cause health inequality by combining the influencing factors of health level. Further, among the many factors that affect health level, which factors contribute more to health inequality? The specific implementation steps included initially analyzed the influencing factors of health level by establishing the demand function of health and estimating the marginal influence coefficient of each factor on the health level:


(2) $$\begin{array}{l} h_{i}=\alpha +\overset{k}{\underset{k=1}{\sum }}\beta_{k}▪x_{ki}+\varepsilon_{i} \end{array}$$

It is assumed here that the corresponding marginal coefficients for each sample are consistent, so it can be inferred that the health differences between individuals are caused by various influencing factors. Substitute Equation (2) into Equation (1):


(3) $$\begin{array}{l} CI\left(h|y\right)=\overset{k}{\underset{k=1}{\sum }}\left(\frac{\beta_{k}▪\bar{x_{k}}}{\bar{h}}\right)▪CI\left(x_{k}|y\right)+\frac{GCI\left(\varepsilon |y\right)}{\bar{h}} \end{array}$$

Where, β_*k*_ is the marginal coefficient of the first *k* factor on health, *x*_*k*_ is the mean value of the first *k* factor, *C*_*k*_ is the CI of the first *k* factor, and $GC_{\varepsilon }=\frac{2}{N}\overset{N}{\underset{i=1}{\sum }}\varepsilon_{i}▪R_{i}$ is the standardized residual CI. As can be seen the Equation (3), CI can be decomposed into two parts, the part is about the explanation variable CI weighting and, after the weight, $\frac{\beta_{k}▪\bar{x_{k}}}{\bar{h}}$ for elastic health level of *x*_*k*_, that is Flexibility_k_, the other part is residual CI and the ratio of average health level, if the health demand function to establish reasonable, residual CI of approximation is 0, had little impact on health inequalities. |C_k_×Flexibility_k_| is the contribution value, and the proportion of contribution value to total contribution value is the contribution rate.

### Data analysis

Data were independently entered twice and validated using Epidata software ver.3.1. The Stata MP.14 and SPSS were used for the data analysis. Descriptive statistical analysis was used to show general information about the participants. According to the formula of the CI, calculated the CI of SAH and the independent variables above. According to the formula of the decomposition of the CI, decomposed the affected factors of SAH. *P* < 0.05 means the difference was statistically significant. Multivariate logistic analysis was used to analyze 12 factors affecting health status. According to the logistic analysis results, backward regression method was used to screen the variables. *P* < 0.10 indicated that the difference was statistically significant.

## Results

### Baseline characteristics

There was a surplus of males over females in both rural-to-urban migrant and urban workers. Among the rural-to-urban migrant workers who participated in the survey, the proportion of those who were married was 67.5%, compared to 43.4% among urban workers. In terms of education level, the proportion of rural-to-urban migrant workers with a high school education or above was 52.1%, lower than the 66.3% of urban workers. In addition, rural-to-urban migrant workers were more likely to have never smoked compared to urban workers (61.4 vs. 49.1%). Regarding health status, 72.7% of rural-to-urban migrant workers self-rated their health as “very good” or “good,” similar to urban workers (73.3%). More information on other characteristics of the rural-to-urban migrant and urban worker participants can be found in Table 1.

**Table 1:** Sociodemographic characteristics of the study participants.

**Variables**	**Rural-to-urban migrant worker (*N* = 1,535)**	**Urban worker (*N* = 523)**	**Overall (*N* = 2,058)**
**Age, year**	36.45 ± 10.57	32.43 ± 9.34	35.43 ± 10.42
**Gender**			
Male	871 (56.7%)	334 (63.9%)	1,205
Female	664 (43.2%)	189 (36.1%)	853
**Marital status**			
Never married	461 (30.0%)	283 (54.1%)	744
Married	1,036 (67.5%)	227 (43.4%)	1,263
Widowed	9 (0.6%)	6 (1.2%)	15
Divorced or separated	29 (1.9%)	7 (1.3%)	36
**Education**			
Elementary school	157 (10.2%)	29 (5.5%)	186
Middle school	578 (37.7%)	147 (28.2%)	725
High school	499 (32.5%)	157 (30.0%)	656
High school above	301 (19.6%)	190 (36.3%)	491
**Income, 10,000 CNY**	4.24 ± 4.45	7.15 ± 7.04	4.98 ± 5.38
**SSRS**	33.79 ± 7.61	28.82 ± 9.42	32.53 ± 8.39
**Exercise**			
Yes	557 (36.3%)	180 (34.4%)	737
No	978 (63.7%)	343 (65.6%)	1,321
**Smoking duration, year**			
Never smoking	942 (61.4%)	257 (49.1%)	1,199
<1	133 (8.7%)	61 (11.7%)	194
(1–5)	217 (14.1%)	148 (28.3%)	365
(5–10)	123 (8.0%)	24 (4.6%)	147
>10	120 (7.8%)	33 (6.3%)	153
**Alcohol consumption, per week, time**			
0	838 (54.6%)	256 (49.0%)	1,094
1	275 (17.9%)	109 (20.8%)	384
2	206 (13.4%)	105 (20.1%)	311
3	107 (7.0%)	29 (5.5%)	136
4	32 (2.1%)	12 (2.3%)	44
≥5	77 (5.0%)	12 (2.3%)	89
**Sleep habits**			
Early to bed and early to wake up	710 (46.2%)	196 (37.5%)	906
Early to bed and late to wake up	96 (6.3%)	68 (13.0%)	164
Late to bed and early to wake up	574 (37.4%)	200 (38.2%)	774
Late to bed and late to wake up	155 (10.1%)	59 (11.3%)	214
**Pension style**			
Children	524 (34.2%)	118 (22.6%)	642
Savings	312 (20.3%)	70 (13.4%)	382
Government	281 (18.3%)	201 (38.4%)	482
Business pension	20 (1.3%)	5 (0.9%)	25
Others	398 (25.9%)	129 (24.7%)	527
**Chronic diseases, number**			
0	1,220 (79.5%)	418 (79.9%)	1,638
1	240 (15.6%)	68 (13.0%)	308
2	62 (4.0%)	33 (6.3%)	95
3	9 (0.6%)	3 (0.6%)	12
≥4	4 (0.3%)	1 (0.2%)	5
**SAH**			
Very good	490 (31.9%)	221 (42.3%)	711
Good	627 (40.8%)	162 (31.0%)	789
Average	391 (25.5%)	132 (25.2%)	523
Poor	24 (1.6%)	6 (1.1%)	30
Very poor	3 (0.2%)	2 (0.4%)	5

SSRS, the Social Support Rating Scale; SAH, self-rated health.

### The concentration index analysis

**Figure 2:**
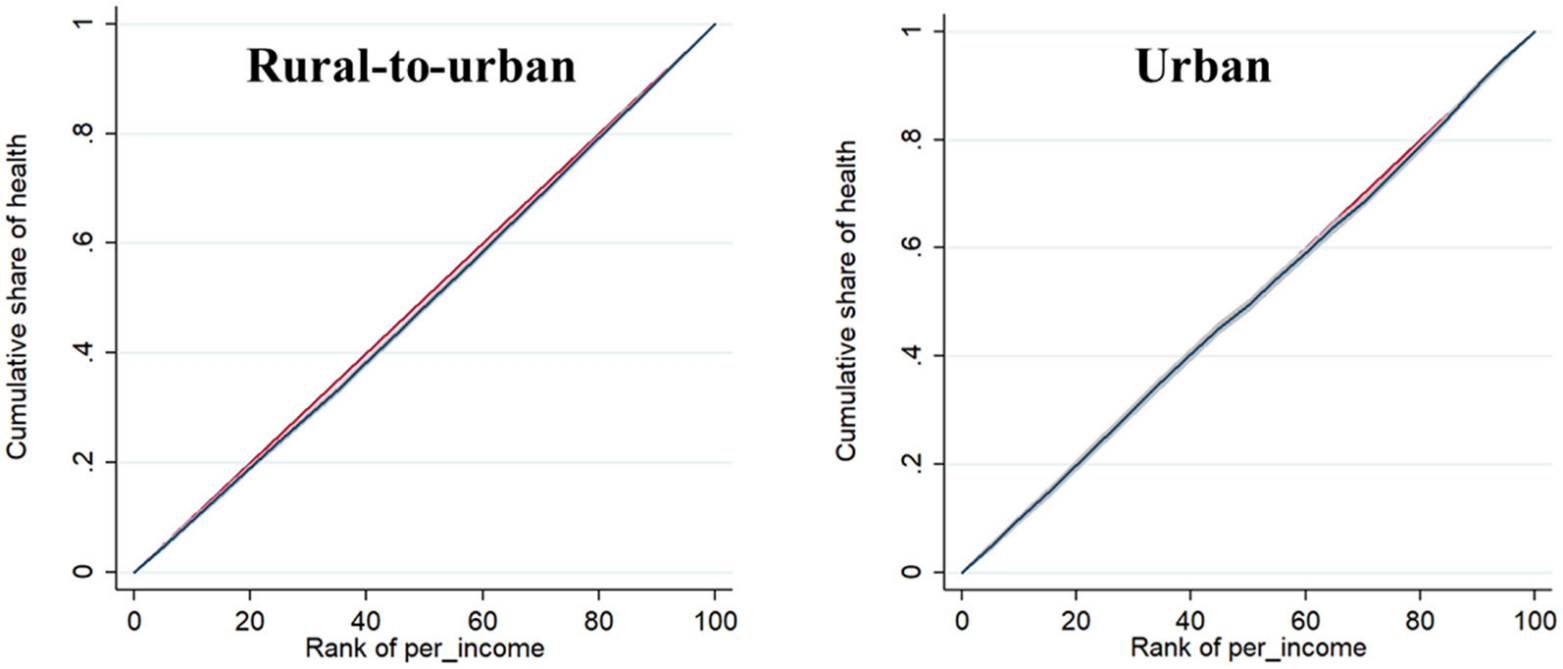
Concentration curve.

**Figure 3:**
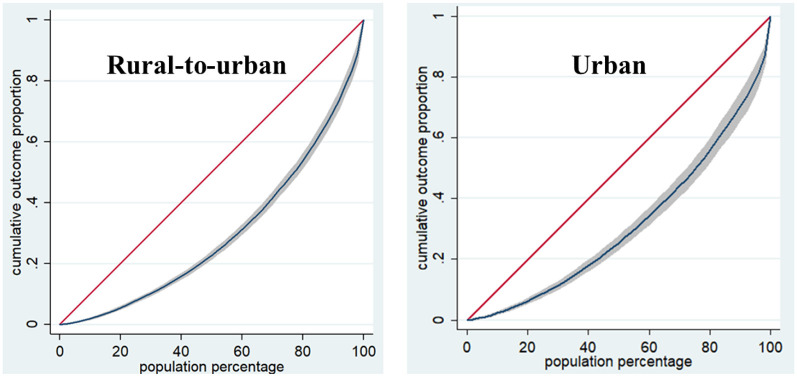
Lorenz curve.

The CI of both health and factors affecting health were calculated by using the concentration index formula in STATA. The CI of health was 0.021 for rural-to-urban migrant workers and 0.005 for urban workers. Figure 2 showed the concentration curve of the two. The Gini coefficient of health was 0.4045 for rural-to-urban migrant workers and 0.3760 for urban workers. Figure 3 showed the Lorenz curve of the two.

Both CIs were >0, the concentration curves were all below the diagonal, which means that both had pro-rich health inequality. The concentration curve of rural-to-urban migrant workers is farther away from the diagonal than that of urban workers, which means that the health inequality of rural-to-urban migrant workers was higher than that of urban workers, and the problem of uneven health distribution was more prominent. The CI of gender was positive for both rural-to-urban migrant and urban workers. The CI of education among rural-to-urban migrant workers was all positive, while among urban workers, all of them were positive, except for middle school, where the CI was negative. In addition, the CI for age, exercise, smoking duration, alcohol consumption, and sleep habits of rural-to-urban migrant workers were positive. The CI for other factors affecting health for rural-to-urban migrant and urban workers was shown in Table 2.

**Table 2:** The concentration index analysis of both health and factors affecting health.

**Variables**	**Rural-to-urban migrant worker**		**Urban worker**	
	**CI**	**SE**	**CI**	**SE**
**Health**	0.021	0.003	0.005	0.099
**Education**				
Elementary school	—	—	—	—
Middle school	0.019	0.005	0.011	0.001
High school	0.020	0.005	0.007	0.022
High school above	0.009	0.006	0.008	0.055
**Gender**				
Female	—	—	—	—
Male	0.021	0.005	0.009	0.036
**Age, year**				
(18, 25)	—	—	—	—
(25, 35)	0.015	0.005	0.007	0.445
(35, 45)	0.017	0.006	0.013	0.745
(45, 71)	0.021	0.006	0.012	0.583
**Exercise**				
No	—	—	—	—
Yes	0.021	0.005	0.010	0.549
**Smoking duration, year**				
Never smoking	—	—	—	—
<1	0.041	0.009	0.016	0.456
(1–5)	0.005	0.007	0.011	0.000
(5–10)	0.035	0.011	0.021	0.939
>10	0.022	0.011	0.017	0.736
**Alcohol consumption, per week, time**				
0	—	—	—	—
1	0.025	0.007	0.011	0.002
2	0.021	0.008	0.011	0.001
3	0.011	0.010	0.022	0.301
4	0.043	0.020	0.020	0.765
≥5	0.013	0.015	0.047	0.164
**Sleep habits**				
Late to bed and late to wake up	—	—	—	—
Late to bed and early to wake up	0.025	0.005	0.009	0.283
Early to bed and late to wake up	0.018	0.012	0.013	0.784
Early to bed and early to wake up	0.018	0.004	0.007	0.000
**Marital status**				
Never married	—	—	—	—
Married	0.021	0.003	0.007	0.815
Widowed	0.063	0.042	0.028	0.326
Divorced or separated	−0.004	0.023	0.044	0.522
**Pension style**				
Children	—	—	—	—
Savings	0.017	0.006	0.012	0.354
Government	0.024	0.006	0.009	0.000
Business pension	−0.001	0.027	0.025	0.559
Others	0.021	0.006	0.010	0.356
**Chronic diseases, number**				
0	—	—	—	—
1	0.017	0.008	0.015	0.420
2	0.001	0.016	0.027	0.118
3	0.089	0.059	0.096	0.667
≥4	−0.019	0.100	…	…
**SSRS**				
Poor	—	—	—	—
Moderate	0.020	0.003	0.0001	0.006
Adequate	0.023	0.012	0.034	0.017

CI, concentration index; SE, standard error; SSRS, the Social Support Rating Scale.

### Decomposition analysis

The results of the decomposition of CI were presented in Table 3. The most important factor causing health inequality among rural-to-urban migrant and urban workers was income, and the contribution of income to health inequality was greater among rural-to-urban migrant workers compared to urban workers (52.24 vs. 32.77%). Exercise (12.98%) and age (10.96%) explained most of the remaining health inequalities among rural-to-urban migrant workers. In contrast, the remaining health inequalities among urban workers were mainly mediated by the number of chronic diseases (14.79%), social support (14.20%), and education (10.57%). The results of multivariate logistic regression analysis were shown in Table 4.

**Table 3:** Decomposition of health inequality of rural-to-urban migrant and urban workers.

**Variables**	**Rural-to-urban migrant worker**			**Urban worker**		
	**C**_K_	**Flexibility**	**Contribution**	**C**_K_	**Flexibility**	**Contribution**
Income	0.4046	0.0196	0.5224	0.3760	0.0178	0.3277
Education	0.0561	0.0150	0.0553	0.0339	0.0638	0.1057
Gender	−0.0167	−0.0113	0.0124	−0.0448	−0.0085	0.0185
Age	−0.0127	−0.1313	0.1096	−0.0199	−0.0613	0.0596
Exercise	0.0525	0.0375	0.1298	0.0371	0.0494	0.0896
Smoking duration	−0.0148	0.0039	0.0039	0.0218	0.0201	0.0213
Alcohol consumption	−0.0011	0.0203	0.0015	−0.0173	0.0385	0.0325
Sleep habits	0.0102	−0.0435	0.0293	0.0030	−0.1259	0.0182
Marital status	−0.0083	0.0192	0.0105	−0.0197	−0.0121	0.0116
Pension style	0.0099	−0.0028	0.0019	0.0117	−0.0446	0.0254
Chronic diseases	−0.0601	−0.0175	0.0693	−0.1799	−0.0168	0.1479
SSRS	0.0049	0.1682	0.0542	−0.0324	0.0898	0.1420

SSRS, the Social Support Rating Scale.

**Table 4:** Multivariate logistic regression analysis.

**Factors**	**Rural-to-urban**					**Factors**	**Urban**				
	**Regression coefficient**	**Standard error**	***P***	**OR**	**95% CI**		**Regression coefficient**	**Standard error**	***P***	**OR**	**95% CI**
Income	−0.01	0.01	0.07	0.99	[0.96, 1.01]	Income	−0.04	0.02	0.05	0.97	[0.93, 1.00]
Education	0.15	0.08	0.04	1.17	[1.01, 1.35]	Education	0.30	0.14	0.03	1.35	[1.03, 1.76]
Age	0.03	0.01	0.00	1.03	[1.02, 1.05]	Age	0.04	0.02	0.02	1.04	[1.01, 1.07]
Alcohol consumption	−0.10	0.05	0.04	0.90	[0.82, 1.00]	Smoking duration	−0.19	0.11	0.07	0.83	[0.67, 1.02]
Sleep habits	0.13	0.06	0.03	1.13	[1.01, 1.27]	Sleep habits	0.23	0.11	0.04	1.25	[1.01, 1.56]
Chronic diseases	0.40	0.13	0.00	1.49	[1.17, 1.91]						

## Discussion

At present, there are few studies on the health inequality of rural-to-urban migrant workers and urban workers in China (34, 35). This study investigated and compared the health inequalities of rural-to-urban migrant workers and urban workers from the same community or workplace. The results show that both rural-to-urban migrant workers and urban workers have health inequalities that are beneficial to the rich. Moreover, compared with the health inequality of urban workers, the health inequality of rural-to-urban migrant workers is more serious. This is consistent with the results of Shao et al.'s study, which shows that there is a serious health inequality among Chinese rural-to-urban migrant workers (8).

The concentration index analysis shows that both urban workers and rural-to-urban migrant workers have health inequalities that are beneficial to the rich, and the health inequality of rural-to-urban migrant workers is higher than that of urban workers. This study found that the highly educated were concentrated in the higher-income groups. As migration in China was mainly from rural-to-urban environments, most rural-to-urban migrant and urban workers will work in jobs with low education and low wages, such as the construction industry or the service sector (8, 36). Those with high levels of education were more likely to work in brain-related jobs, which would be more financially rewarding. At the same time, there are gender-related income differences between rural-to-urban migrant and urban workers, with males being more concentrated in higher income groups compared to females, which was consistent with previous research (8, 37). The studies revealed that female workers, especially rural-to-urban migrant workers, were more likely to be disadvantaged. We found an interesting phenomenon that when the number of chronic disease cases is 2–3, urban workers have more serious health inequalities than rural-to-urban migrant workers, which is beneficial to the rich. This is contrary to the research results of Li and Tang (38). The reason for this result may be due to the weak health awareness of rural-to-urban migrant workers, they believe that physical examination will increase their unnecessary costs, so only when the body has obvious symptoms, they will go to the hospital. This leads to a low self-report rate of chronic diseases among rural-to-urban migrant workers (39). Secondly, the treatment of chronic diseases requires long-term and sustained economic expenditure, which gives urban workers with higher economic levels more opportunities to obtain treatment (40). This means that we should not only intervene in the health inequality of rural-to-urban migrant workers from the perspective of income, but also pay attention to the accessibility of basic public services for rural-to-urban migrant workers and whether they can obtain basic medical resources. In addition, we also found that when the SSRS score is 45–66, urban workers have more serious health inequalities than rural-to-urban migrant workers. This is contrary to the results of a previous study (28). The explanation of the possibility is that rural-to-urban migrant workers rely more on relatives and friends than money to obtain medical resource information in a strange urban environment. Urban workers with low economic level have less access to social support, so their health inequality is more serious.

There was currently a lot of research looking at the impact of income on health inequality (3, 5, 12). However, compared with Li et al. (3) who divided the income level of rural-to-urban migrant workers into four levels, this study adopted the specific amount of income for quantitative analysis, so as to more objectively and accurately reflect the impact of income on health inequality. The results of the decomposition of health inequalities suggested that income was the most important factor contributing to health inequalities among rural-to-urban migrant workers and urban workers, which was consistent with other studies (8, 41, 42). Because income determines the living environment, nutritional conditions, and health resources available to both rural-to-urban migrant workers and urban workers, income disparities can lead to significant health inequalities. Since both the CI of health and the flexibility were positive, an increase in income can contribute significantly to better health, but the resulting income disparity can lead to increasing health disparities among individuals. To reduce income disparity as a sensible option to reduce health inequality, relevant authorities should continuously optimize the income distribution system for workers, especially rural-to-urban migrant workers, and increase the regulation of income distribution, so as to reduce income disparity and alleviate health inequality (8, 43). In addition, exercise (12.98%), age (10.96%), and the number of chronic diseases (6.93%) were important sources of health inequality for rural-to-urban migrant workers. Stalsberg et al.'s study found that the only consistent relationship between social economic status (SES) and self-reported physical activity is physical activity in recreational or leisure time (44). The results of a study also support this view, which shows that differences in sports infrastructure and public resources available to different income groups can also cause unfair health levels (45). It is crucial for social organizations and governments to strengthen the construction of basic sports equipment and improve the physical activity of rural-to-urban migrant workers. According to the survey, 49.5% of rural older adults live in low-income families (46). With the increase of age, the source of medical expenditure of low-income rural-to-urban migrant workers is more children, so the medical resources obtained by low-income rural-to-urban migrant workers are more limited than those of high-income rural-to-urban migrant workers. In addition, the social population aging model has gradually shifted to the disease model, which is mainly reflected in chronic diseases and disabilities and more complex health conditions. The treatment of chronic diseases requires long-term medical expenditure, which is also known as “wealthy diseases” (47). Su et al.'s research results show that higher income groups have better health services, which is the same as our research results (48). This supports the active call for interventions to reduce the health costs of urbanization of rural-to-urban migrant workers. However, the number of chronic diseases (14.79%), and social support (14.20%) contributed to the majority of health inequalities among urban workers. The reasons for this result may be the following aspects. Firstly, because the treatment of chronic diseases requires long-term use of drugs, some diseases may affect the work of urban workers and thus reduce economic income. Compared with urban workers with lower economic level, wealthy urban workers have stronger ability to resist disease risk and can obtain more medical resources due to their higher income and deposits (40). Therefore, it cannot be ignored to promote health equity by strengthening public health and health knowledge education, carrying out health lectures, and improving the medical insurance system. Secondly, a study shows that the individual's health level is related to the quantity and quality of social support, and social support can directly affect the individual's physical and mental health (49). Urban workers with higher social support tend to have higher income levels than urban workers with lower income levels, and provide financial help in a timely manner when their relatives have health problems. Thirdly, studies have shown that people with lower levels of education generally earn lower wages by engaging in simple repetitive labor work. Their working environment is poor, and there are more unhealthy factors. Due to the lack of awareness of health care, they cannot release the pressure of long-term work through physical activity, which further damages their health (50). Therefore, community hospitals can improve the accessibility of health education and formulate education content suitable for urban workers with low education level, so as to reduce the health inequality caused by education.

The present study has the following limitations. First, this study was a cross-sectional study, but individual health levels are a dynamic process, so a longitudinal study design seems to provide a better understanding of trends in individual health status over time, and thus a more comprehensive understanding of the factors affecting workers' health inequalities. Second, the data in this study were collected from five coastal cities with a concentration of Chinese workers, which may reflect the health inequalities of workers in the eastern coastal region, but is not representative of other regions. Additionally, although we try to select participants as randomly as possible during the sampling phase, it was still difficult to ensure that there was no potential bias. Finally, this study used self-reported health, which may bias the findings to some extent. Researchers can broaden their thinking through this study and conduct a comprehensive and systematic study from the 8 aspects of the SF-8 (Short Form Health Survey) scale. Researchers can also use an objective and accurate evaluation method to explore the health inequality of rural-to-urban migrant workers.

The results of this study indicated that significant health inequalities were found among both rural-to-urban migrant and urban workers. In addition, income, exercise, age, and the number of chronic diseases were important sources of health inequality among rural-to-urban migrant workers; while income, the number of chronic diseases, and social support contributed most of the health inequality among urban workers. The government and relevant authorities should formulate timely policies and adopt targeted measures to improve health inequalities.

## Data availability statement

The original contributions presented in the study are included in the article/supplementary material, further inquiries can be directed to the corresponding author.

## Ethics statement

The studies involving humans were approved by Ethics Committee of Wenzhou Medical University. The studies were conducted in accordance with the local legislation and institutional requirements. The participants provided their written informed consent to participate in this study.

## Author contributions

SD: Conceptualization, Formal analysis, Methodology, Writing—original draft, Writing—review & editing. YY: Conceptualization, Data curation, Formal analysis, Writing—original draft, Writing—review & editing. MZ: Investigation, Writing—original draft, Writing—review & editing. HZ: Investigation, Writing—original draft, Writing—review & editing. TL: Writing—original draft, Writing—review & editing. FC: Conceptualization, Supervision, Validation, Visualization, Writing—original draft, Writing—review & editing.

## References

[1] Statistics NBo. Population Mortality and Evaluation of Life Expectancy. (2020). Available online at: https://www.stats.gov.cn/sj/ndsj/2020/indexch.htm (accessed April 22, 2024).

[2] CPC Central Committee. Issued BY the CPC Central Committee and The State Council “Healthy China 2030” Plan Outline. (2016). Available online at: http://cpc.people.com.cn/n1/2016/1026/c64387-28807482.html (accessed April 22, 2024).

[3] Li D Zhou Z Shen C Zhang J Yang W Nawaz R. Health disparity between the older rural-to-urban migrant workers and their rural counterparts in China. Int J Environ Res Public Health. (2020) 17:30955. 10.3390/ijerph17030955 32033086 PMC7038012

[4] Liang D Teng M Xu D. Impact of perceived social support on depression in Chinese rural-to-urban migrants: the mediating effects of loneliness and resilience. J Community Psychol. (2019) 47:1603–13. 10.1002/jcop.22215 31332801

[5] Pham KTH Nguyen LH Vuong QH Ho MT Vuong TT Nguyen HT. Health inequality between migrant and non-migrant workers in an industrial zone of Vietnam. Int J Environ Res Public Health. (2019) 16:91502. 10.3390/ijerph16091502 31035337 PMC6539052

[6] Programme UND. UNDP Support to the Implementation of the 2030 Agenda for Sustainable Development. (2016). Available online at: https://www.undp.org/publications/undp-support-implementation-2030-agenda (accessed April 22, 2024).

[7] Fan C Ouyang W Tian L Song Y Miao W. Elderly health inequality in China and its determinants: a geographical perspective. Int J Environ Res Public Health. (2019) 16:162953. 10.3390/ijerph16162953 31426371 PMC6719074

[8] Shao C Meng X Cui S Wang J Li C. Income-related health inequality of migrant workers in China and its decomposition: an analysis based on the 2012 China Labor-force Dynamics Survey data. J Chin Med Assoc. (2016) 79:531–7. 10.1016/j.jcma.2016.02.009 27288189

[9] Lin D Li X Wang B Hong Y Fang X Qin X. Discrimination, perceived social inequity, and mental health among rural-to-urban migrants in China. Community Ment Health J. (2011) 47:171–80. 10.1007/s10597-009-9278-4 20033772 PMC2891847

[10] Bor J Cohen GH Galea S. Population health in an era of rising income inequality: USA, 1980-2015. Lancet. (2017) 389:1475–90. 10.1016/S0140-6736(17)30571-8 28402829

[11] Wagstaff A Paci P van Doorslaer E. On the measurement of inequalities in health. Soc Sci Med. (1991) 33:545–57. 10.1016/0277-9536(91)90212-U 1962226

[12] Li D Zhai S Zhang J Yang J Wang X. Assessing income-related inequality on health service utilization among Chinese rural migrant workers with new co-operative medical scheme: a multilevel approach. Int J Environ Res Public Health. (2021) 18:2010851. 10.3390/ijerph182010851 34682615 PMC8535776

[13] Barreto ML. Health inequalities: a global perspective. Cien Saude Colet. (2017) 22:2097–108. 10.1590/1413-81232017227.02742017 28723991

[14] Eozenou PH Neelsen S Lindelow M. Child health outcome inequalities in low and middle income countries. Health Syst Reform. (2021) 7:e1934955. 10.1080/23288604.2021.1934955 34402412

[15] Silva-Peñaherrera M Lopez-Ruiz M Merino-Salazar P Gómez-García AR Benavides FG. Health inequity in workers of Latin America and the Caribbean. Int J Equity Health. (2020) 19:109. 10.1186/s12939-020-01228-x 32611402 PMC7329389

[16] Pascual M Cantarero D Lanza P. Health polarization and inequalities across Europe: an empirical approach. Eur J Health Econ. (2018) 19:1039–51. 10.1007/s10198-018-0997-8 30066237

[17] Hosseinpoor AR Bergen N Schlotheuber A. Promoting health equity: WHO health inequality monitoring at global and national levels. Glob Health Action. (2015) 8:29034. 10.3402/gha.v8.29034 26387506 PMC4576419

[18] Li X Stanton B Fang X Xiong Q Yu S Lin D. Mental health symptoms among rural-to-urban migrants in China: a comparison with their urban and rural counterparts. World Health Popul. (2009) 11:24–38. 10.12927/whp.2009.20868 20039592

[19] Yang Y Chen B Huang P Wang Y Zhang L Cai F. Prevalence and influencing factors of depressive symptoms among rural-to-urban migrant workers in China: a systematic review and meta-analysis. J Affect Disord. (2022) 307:11–9. 10.1016/j.jad.2022.03.061 35351493

[20] Lau JT Cheng Y Gu J Zhou R Yu C Holroyd E. Suicides in a mega-size factory in China: poor mental health among young migrant workers in China. Occup Environ Med. (2012) 69:526. 10.1136/oemed-2011-100593 22374180

[21] Ma S Li Q Zhou X Cao W Jiang M Li L. Assessment of health inequality between urban-to-urban and rural-to-urban migrant older adults in China: a cross-sectional study. BMC Public Health. (2020) 20:268. 10.1186/s12889-020-8341-5 32093668 PMC7041246

[22] China NBoSo. 2022 Migrant Worker Monitoring Survey Report. (2023). Available online at: https://www.stats.gov.cn/xxgk/sjfb/zxfb2020/202304/t20230428_1939125.html (accessed April 22, 2024).

[23] Alam MA-U He X. Is the discrimination against migrant workers tending toward zero in urban China? Int Stud Econ. (2022) 17:65–81. 10.1002/ise3.4

[24] Mohabir N Jiang Y Ma R. Chinese floating migrants: rural-urban migrant labourers' intentions to stay or return. Habitat Int. (2017) 60:101–10. 10.1016/j.habitatint.2016.12.008

[25] Zhao X. Migrants and urban wage: evidence from China's internal migration. China Econ Rev. (2020) 61:101287. 10.1016/j.chieco.2019.03.006

[26] Bradley SH. The ethics and politics of addressing health inequalities. Clin Med. (2021) 21:147–9. 10.7861/clinmed.2020-0945 33762377 PMC8002784

[27] Thomeer MB Yahirun J Colón-López A. How families matter for health inequality during the COVID-19 pandemic. J Fam Theory Rev. (2020) 12:448–63. 10.1111/jftr.12398 33841554 PMC8034594

[28] Yang Y Zhao S Lin L Qian J Zhang H Cai F. Social support and quality of life in migrant workers: focusing on the mediating effect of healthy lifestyle. Front Public Health. (2023) 11:1061579. 10.3389/fpubh.2023.1061579 37033034 PMC10076876

[29] Sun J Sun R Jiang Y Chen X Li Z Ma Z. The relationship between psychological health and social support: evidence from physicians in China. PLoS ONE. (2020) 15:e0228152. 10.1371/journal.pone.0228152 31995601 PMC6988930

[30] Zhang C Zhao H Zhu R Lu J Hou L Yang XY. Improvement of social support in empty-nest elderly: results from an intervention study based on the Self-Mutual-Group model. J Public Health. (2019) 41:830–9. 10.1093/pubmed/fdy185 30428059

[31] Au N Johnston DW. Self-assessed health: what does it mean and what does it hide? Soc Sci Med. (2014) 121:21–8. 10.1016/j.socscimed.2014.10.007 25306406

[32] Wagstaff A Doorslaer EV Watanabe N. On decomposing the causes of health sector inequalities with an application to malnutrition inequalities in Vietnam. J Econom. (2003) 112:207–23. 10.1016/S0304-4076(02)00161-6

[33] Xu Y Zhu S Zhang T Wang D Hu J Gao J. Explaining income-related inequalities in dietary knowledge: evidence from the China Health and Nutrition Survey. Int J Environ Res Public Health. (2020) 17:20532. 10.3390/ijerph17020532 31952113 PMC7013705

[34] Qin L Chen CP Liu X Wang C Jiang Z. Health status and earnings of migrant workers from rural China. China World Econ. (2015) 2:16. 10.1111/cwe.12108

[35] Bener A. Health status and working condition of migrant workers: major public health problems. Int J Prev Med. (2017) 8:68. 10.4103/ijpvm.IJPVM_396_16 28966757 PMC5609361

[36] Zhang L Sharpe RV Li S Darity WA. Wage differentials between urban and rural-urban migrant workers in China. China Econ Rev. (2016) 41:222–33. 10.1016/j.chieco.2016.10.004

[37] Yang P Zhang G. An empirical analysis of the gender wage differential of migrant workers in China based on the improved brown decomposition approach. J Guangdong Univ Fin Econ. (2012) 27:74–83.

[38] Li C Tang C. Income-related health inequality among rural residents in western China. Front Public Health. (2022) 10:1065808. 10.3389/fpubh.2022.1065808 36589999 PMC9797679

[39] Fu D Liu L Zhang X Yu C Luo H Li N. The relationship between urban and rural health insurance and the self-rated health of migrant workers in Southwest China. BMC Health Serv Res. (2021) 21:614. 10.1186/s12913-021-06646-3 34182997 PMC8240306

[40] Allain S Naouri D Deroyon T Costemalle V Hazo JB. Income and professional inequalities in chronic diseases: prevalence and incidence in France. Public Health. (2024) 228:55–64. 10.1016/j.puhe.2023.12.022 38306754

[41] Leng L. Decomposing wage differentials between migrant workers and urban workers in urban China's labor markets. China Econ Rev. (2012) 23:461–70. 10.1016/j.chieco.2012.03.004

[42] Peng Y Chang W Zhou H Hu H Liang W. Factors associated with health-seeking behavior among migrant workers in Beijing, China. BMC Health Serv Res. (2010) 10:69. 10.1186/1472-6963-10-69 20298613 PMC2848137

[43] Chen J. Health Inequality between Urban and Rural Residents in China: Evidence from CFPS2012 and CFPS2016 Data (Master thesis). Central China Normal University, Wuhan, China (2019).

[44] Stalsberg R Pedersen AV. Are differences in physical activity across socioeconomic groups associated with choice of physical activity variables to report? Int J Environ Res Public Health. (2018) 15:50922. 10.3390/ijerph15050922 29734745 PMC5981961

[45] Sfm C Van Cauwenberg J Maenhout L Cardon G Lambert EV Van Dyck D. Inequality in physical activity, global trends by income inequality and gender in adults. Int J Behav Nutr Phys Act. (2020) 17:142. 10.1186/s12966-020-01039-x 33239036 PMC7690175

[46] Guo B Xie X Wu Q Zhang X Cheng H Tao S. Inequality in the health services utilization in rural and urban china: a horizontal inequality analysis. Medicine. (2020) 99:e18625. 10.1097/MD.0000000000018625 31914043 PMC6959938

[47] Shang XT Wei ZH. Socio-economic inequalities in health among older adults in China. Public Health. (2023) 214:146–52. 10.1016/j.puhe.2022.11.013 36549024

[48] Su B Li D Xie J Wang Y Wu X Li J. Chronic disease in China: geographic and socioeconomic determinants among persons aged 60 and older. J Am Med Dir Assoc. (2023) 24:206–12.e5. 10.1016/j.jamda.2022.10.002 36370750

[49] Christakis NA. Social networks and collateral health effects. Br Med J. (2004) 329:184–5. 10.1136/bmj.329.7459.184 15271805 PMC487721

[50] Hui H. The influence mechanism of education on health from the sustainable development perspective. J Environ Public Health. (2022) 2022:7134981. 10.1155/2022/7134981 35910750 PMC9328960

